# It’s a Matter of Mind! Cognitive Functioning Predicts the Athletic Performance in Ultra-Marathon Runners

**DOI:** 10.1371/journal.pone.0132943

**Published:** 2015-07-14

**Authors:** Giorgia Cona, Annachiara Cavazzana, Antonio Paoli, Giuseppe Marcolin, Alessandro Grainer, Patrizia Silvia Bisiacchi

**Affiliations:** 1 Department of General Psychology, University of Padua, Padua, Italy; 2 Department of Neuroscience, University of Padua, Padua, Italy; 3 Department of Biomedical Science, University of Padua, Padua, Italy; 4 Center for Cognitive Neuroscience, University of Padua, Padua, Italy; University of Bologna, ITALY

## Abstract

The present study was aimed at exploring the influence of cognitive processes on performance in ultra-marathon runners, providing an overview of the cognitive aspects that characterize outstanding runners. Thirty runners were administered a battery of computerized tests right before their participation in an ultra-marathon. Then, they were split according to the race rank into two groups (i.e., faster runners and slower runners) and their cognitive performance was compared. Faster runners outperformed slower runners in trials requiring motor inhibition and were more effective at performing two tasks together, successfully suppressing the activation of the information for one of the tasks when was not relevant. Furthermore, slower runners took longer to remember to execute pre-defined actions associated with emotional stimuli when such stimuli were presented. These findings suggest that cognitive factors play a key role in running an ultra-marathon. Indeed, if compared with slower runners, faster runners seem to have a better inhibitory control, showing superior ability not only to inhibit motor response but also to suppress processing of irrelevant information. Their cognitive performance also appears to be less influenced by emotional stimuli. This research opens new directions towards understanding which kinds of cognitive and emotional factors can discriminate talented runners from less outstanding runners.

## Introduction

“I just run. I run in a void. Or maybe I should put it the other way: I run in order to acquire a void. But as you might expect, an occasional thought will slip into this void. People’s minds can’t be a complete blank. Human beings’ emotions are not strong or consistent enough to sustain a vacuum. What I mean is, the kinds of thoughts and ideas that invade my emotions as I run remain subordinate to that void.”

Haruki Murakami—What I Talk About When I Talk About Running

All kinds of sports imply, to different extents, the application of cognitive, perceptual and motor skill [1, 2]. Nevertheless, although superior performance is clearly evident on observation, the cognitive mechanisms that contribute to a successful performance are less clear. For several decades researchers have sought to better understand the cognitive factors that are able to discriminate between talents and less outstanding athletes [3]. Outstanding athletes were shown to have enhanced declarative and procedural knowledge, and to be more able at making decisions and at extrapolating relevant information from the environment to anticipate future events and outcomes [4–6]. Experts seem also to have a more effective visuo-spatial processing and greater selective attention [7–9]. In particular, the effect of focus of attention on athletic skills has been extensively explored across sporting domains. Attentional focus is typically classified as internal or external, where the internal focus is meant to be directed toward the performance of movements, whereas the external one is meant to direct attention toward the effects of a movement [10] and/or to external environmental stimuli [11]. Overall, an external focus of attention appears to be more beneficial for a successful sporting performance [12, 13].

Furthermore, motor response selection and inhibition processes were shown to be crucial, for example, in fencing, baseball, tennis and soccer [14–17].

Despite the increasing evidence of the key role of cognitive factors across a wide range of sporting domains, the contribution of such factors to endurance sports and, more specifically, to running performance, is still poorly understood. The few studies that have addressed this issue focused on the influence of cognitive strategies and focus of attention on quality and performance of the run. Cognitive strategies are typically subdivided into associative strategies, which imply directing of attention towards task-relevant stimuli and physiological sensations experienced during exercise, and dissociative strategies, consisting in directing attention toward distracting thoughts, as work, relationships, and other kinds of thoughts unrelated to the experience of running [18]. Generally, these studies showed that runners adopting an associative strategy ran faster than runners adopting a dissociative strategy [19; 18]. A recent study also highlighted that having an external focus of attention increases running economy (measured as oxygen consumption at a set running speed), leading to a better performance as compared with an internal focus of attention [20].

Since the contribution of the other cognitive aspects has been almost neglected, the present study aimed to provide, for the first time, an overview of the impact of various cognitive functions upon running performance. More specifically, the starting point questions were: Could cognitive functioning contribute to running performance? And, if so, which cognitive processes are the best mediators of running performance? To answer these questions we asked a group of ultra-runners to execute a series of cognitive tasks immediately before the running race. Then, we analysed the cognitive performance on these tasks comparing the runners who obtained a batter rank in the race (i.e., faster runners) with those who obtained a worse rank (i.e., slower runners).

We explored cognitive functioning by means of the modified versions of two computerized tasks: The Inhibitory Control Task (ICT) and a dual-task paradigm with emotional stimuli, which have been already utilized in our lab [21–25]. The ICT is composed of multiple types of trials, thus allowed us to test distinct cognitive processes, including response speed, selective attention, working memory updating and response inhibition [24]

**Fig 1:**
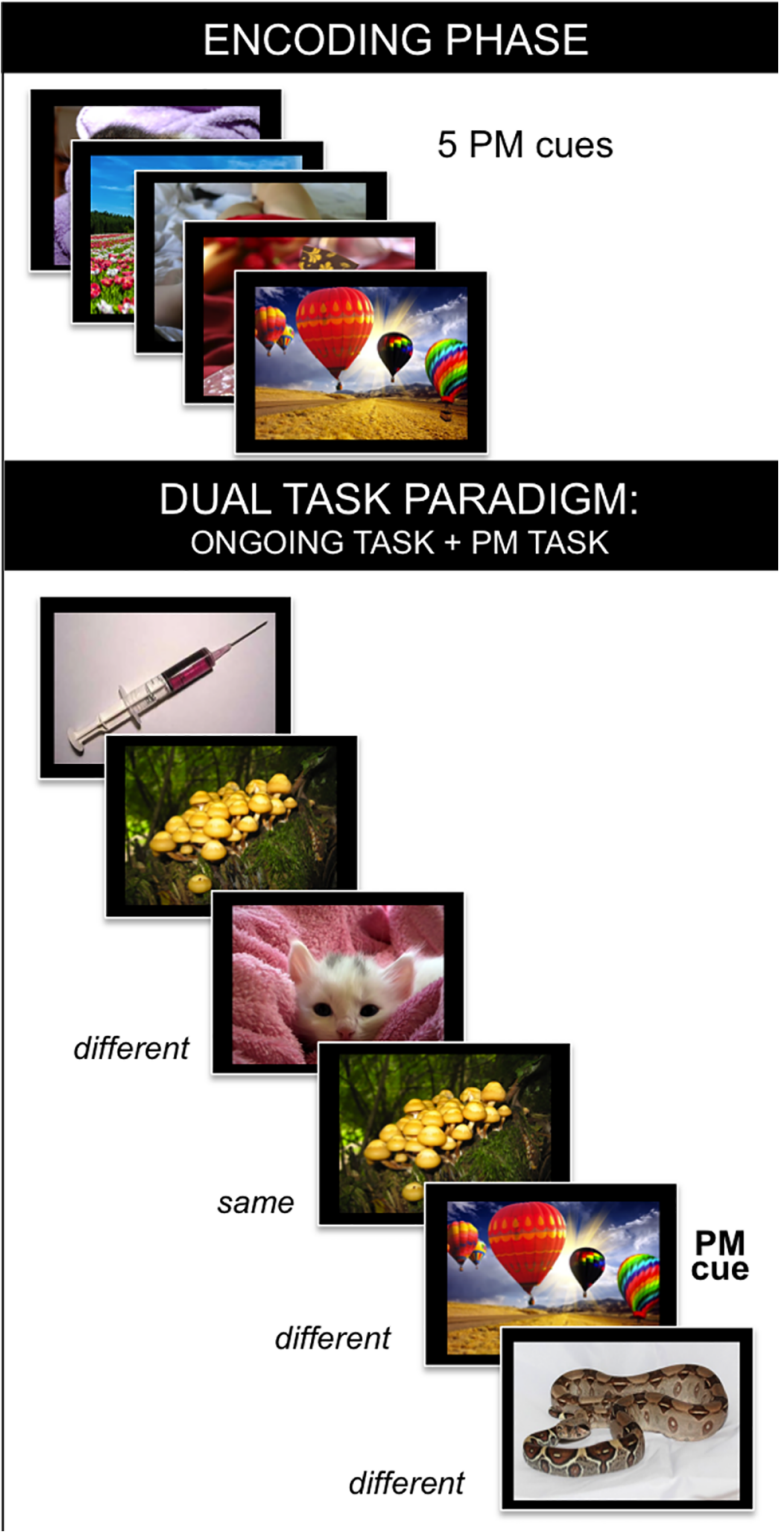
Schematic illustration of the Dual-Task paradigm. The figure illustrates the pleasant PM session, in which five pleasant PM cues needed to be encoded for later execution of the intention. The same tasks and procedure were run for both unpleasant and neutral sessions. Although not displayed, a blank screen with a fixation cross (lasting 1200, 1400, or 1600 ms) always occurred between two distinct stimuli. For the ongoing task, participants had to press one of two keys with the right hand to decide whether the picture was same or different from the picture presented two trials before. For the PM task, participants were required to remember to press an additional key, with their left index finger, when they saw a picture presented during the encoding phase. *Note*: *The pictures displayed in the figure are not those used in the study*, *but are taken from Internet only for illustration purposes*.

To better explore the impact of executive functions on running, we utilized a dual-task paradigm, in which two distinct tasks, heavily dependent on frontal executive processes, needed to be executed simultaneously (Fig 1). The dual-task paradigm consists of an ongoing activity, namely a working memory 2-back task, and a Prospective Memory (PM) task [25]. For the 2-back task, participants were instructed to decide whether the picture occurring on the screen was same or different from the picture presented two trials before by pressing one of two possible response keys (i.e., 2-back task). While executing the ongoing task, they had to remember to complete a pre-specified intention (i.e., pressing a third key) when a pre-memorized picture, namely the PM cue, occurred on the screen amid the ongoing trials (i.e., PM task). Therefore, by using such paradigm we were able to test the runners’ ability: (*i*) to manage two tasks simultaneously; (*ii*) to monitor the external, ongoing, stimuli driven by an internal goal (i.e., the identification of the PM cue), providing information about the runners’ attitude to adopt an internal *versus* external focus of attention, as postulated in the PM context by the Attention to Delayed Intention (AtoDI) model [26]; (*iii*) to remember to execute delayed intentions when the appropriate cues occur; (*iv*) to process and react to emotional stimuli. In order to address the fourth issue, we included pictures that were characterized by a specific emotional valence (i.e., pleasant, unpleasant, or neutral) in both the ongoing and PM tasks. Cognitive-evaluative reactions to emotional stimuli and situations were shown, indeed, to be pivotal for athletic performance [27–29]. Thus, the inclusion of emotional stimuli in this paradigm was important to illuminate the relationship between processing and reacting to emotional stimuli and the subsequent running behaviour.

## Materials and Methods

### Running race and Participants

Data were collected on July 25^th^ 2014, in occasion of the Trans d’Havet race. This competition took place in the northeast of Italy and was part of the Ultra race of the European Skyrunning championships. The track consisted of 80 km with a total elevation of 5500 mt and a maximum altitude of 2238 mt. The race started on Saturday at 12.00 pm. The organizations guaranteed medical stations and rest stops with drinks and food along the whole race. Each participant had to pass pre-defined gate not exceeding a certain time to continue the race. Unexpectedly, the race was interrupted because of weather conditions, and the race rank was obtained from the order of arrival recorded at the last pre-defined gate passed before the interruption of the race, which corresponded to the 30^th^ km for all the participants.

Thirty ultra-marathon runners (*M* = 43 years, S.D. = 8.6) took part in this study. The determination of the sample size to detect a medium size effect (*η*_*p*_ *²* = .25) was based on a previous study that used the same task (i.e., the ICT; [23]).

All participants were males, had normal, or corrected-to-normal, vision and no neurological, psychiatric or psychological (including phobias) pathologies. Participants were in good physical health, as proven by the medical certificate. All the runners were tested on cognitive tasks right before their participation to the ultra-marathon (between the 7 pm and the 9 pm). Then, a median split based on the ranking recorded at the last pre-defined gate before the interruption of the race was performed. This allowed us to create two groups, distinguishing between faster runners and slower runners. The two groups did not differ either in age or educational level (Faster runners: Age 42.8 ± 9.6 yrs, Education 14.0 ± 4.1 yrs; Slower runners: 42.1 ± 7.7 yrs, Education 16.3 ± 2.5 yrs; all ps > .05).

### Ethics Statement

The study was approved by the ethical committee of the Department of Biomedical Sciences (University of Padua) and was conducted according to the principles of the Declaration of Helsinki. All the participants were informed about the experimental procedure and signed a written consent form.

### Inhibitory Control Task

The ICT was adapted from the version used in our previous studies [23, 24]. Black letters were presented, one after the other, for 500 ms without inter-stimulus interval, in the center of a white background computer screen. Interspersed within the other letters, the target letters X and Y were presented. During the first session of the task, the participants were instructed to respond by pressing the spacebar for every X and Y (detect trials). During the second session of the task, participants were instructed to press the spacebar only when X and Y were alternating (go trials) and to inhibit their response when X and Y were repeated (nogo trials). Two target letters never occurred consecutively. The first session comprised 122 distracting letters and 30 target letters (detect trials). The second session was composed of 3 blocks, for a total of 567 distracting letters, 90 go trials and 18 nogo trials.

### Dual-Task paradigm

Following our previous study [25], the dual-task paradigm consisted of an ongoing working memory task and a PM task, simultaneously executed (Fig 1). The ongoing task was a 2-back task comprising pleasant, neutral and unpleasant pictures. Pictures were selected from the International Affective Picture System ([30]; see [25], for more details on the features of the stimuli selected). Participants were instructed to decide whether the picture occurring on the screen was same or different from the picture occurring two trials before by pressing one of two possible response keys on the keyboard with the index or middle finger of their right hand (‘N’ or ‘M’ keys). On each trial, the stimulus remained on the screen for 2000 ms or until a response was made, and was followed by a black screen with a fixation cross that pseudo-randomly lasted 1200, 1400, or 1600 ms. Simultaneously with the ongoing task, individuals were instructed to remember to accomplish a PM task, which consisted in pressing the ‘Z’ key, with their left index finger, when particular pictures (i.e., PM cues) occurred on the screen. The paradigm was composed of three PM sessions, which differ for the emotional valence of the PM cue (pleasant, unpleasant, neutral). Each session was preceded by an encoding phase, during which the PM cues were presented, one after the other, in the center of the screen and participants were required to memorize them. Within a PM session, pleasant, neutral and unpleasant ongoing pictures were pseudo-randomly presented, whereas the valence of the PM cues was constant. The order of the PM sessions was counterbalanced across participants. Each of the PM sessions comprised 55 ongoing stimuli and 5 PM cues each. A PM cue was never also a ‘same’ 2-back trial. Before the PM sessions, a practice block comprising 39 ongoing trials was given.

### Data Analysis

We compared the ICT performance between faster runners *versus* slower runners by analyzing the mean accuracy for the three ICT types of trials (detect, go, nogo trials) and the RTs for the detect and go trials by means of two separate ANOVAs.

In order to investigate the effect of monitoring for emotional PM cues on the ongoing performance, the mean RTs and the proportion of correct responses to the 2-back task were analyzed in two separate ANOVAs including one between-subject factor (i.e., runners group: faster *versus* slower runners) and three within-subject factors: Stimulus type (same 2-back trial, different 2-back trial), PM cue valence (unpleasant, neutral, pleasant) and Ongoing stimulus valence (unpleasant, neutral, pleasant). Indeed, previous studies showed that the allocation of attentional resources towards ongoing stimuli to monitor for PM cue were reflected in an increase of RTs and were greater when there was a match between the valence of the PM cues and the valence of the ongoing stimuli, revealing the Stimulus Specific Interference Effect (SSIE; [25]).

The mean RTs and accuracy in the PM task were entered into two ANOVAs with the Runners group and the Valence of the PM cues as factors.

For all the analyses, post hoc comparisons were conduced applying the Fisher's LSD (Least Significant Difference) correction. We estimated effect sizes using partial eta squared (*η*_*p*_ *²*).

## Results

### Inhibitory Control Task

**Fig 2:**
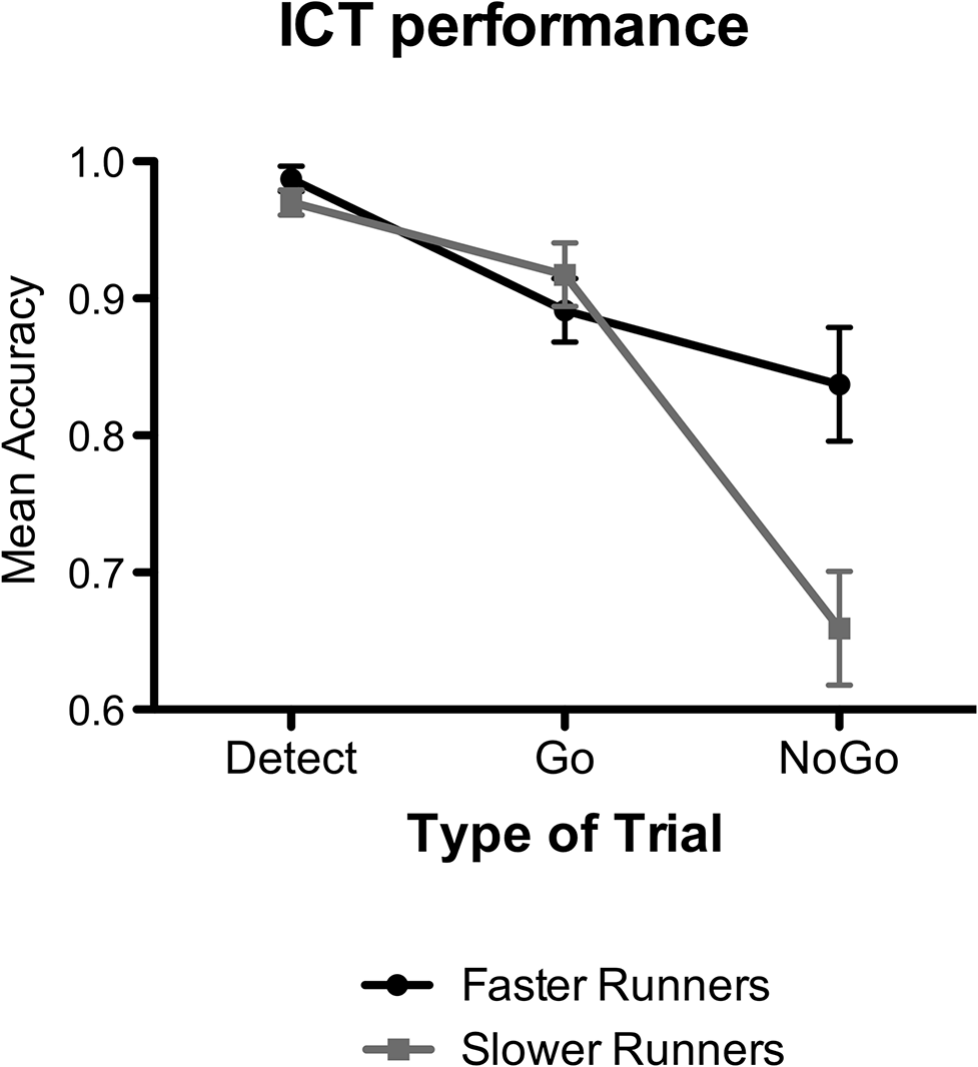
Mean Accuracy in the Inhibitory Control Task (ICT) trials for the faster and slower runners. Faster runners outperform slower runners selectively in the nogo trials, whereas they did not differ from slower runners in the detect and go trials. Vertical bars represent standard error.

The analysis of mean accuracy in the ICT revealed a significant main effect of the Runners group [*F*(1,28) = 4.81, *p* < .05, *η*_*p*_ *²* = .15], of the Type of trial [*F*(2,56) = 40.99, *p* < .001, *η*_*p*_ *²* = .54], as well as a significant interaction between the two variables [*F*(2,56) = 8.57, *p* < .001, *η*_*p*_ *²* = .23]. As can be also seen in Fig 2, post hoc comparisons revealed that faster runners outperformed slower runners selectively in the nogo trials (*p* < .001), whereas did not differ from slower runners in the detect and go trials (*p* > .05).

The same analysis performed on RTs showed no significant difference between the two groups of runners [*F*(1,28) = 0.80, *p* > .05, *η*_*p*_ *²* = .02, Faster runners: *M* = 455 ms, standard error, *SE* = 16.97; Slower runners: *M* = 438 ms, *SE* = 8.90]. It however revealed a main effect of the Type of trial, revealing that the RTs were slower in go trials (*M* = 476 ms, *SE* = 12.47) than in the detect trials (*M* = 417 ms, *SE* = 8.46) [*F*(1,28) = 4.81, *p* < .001, *η*_*p*_ *²* = .57], for both groups.

### Dual-Task Paradigm

The analysis of the RTs in the ongoing task revealed a significant Runners group × PM cue valence interaction [*F*(2,56) = 3.48, *p* < .05, *η*_*p*_ *²* = .11]. Post hoc comparisons showed that, as compared with faster runners, slower runners tended to have increased RTs in ongoing trials when they had to monitor for pleasant and unpleasant PM cues (both *ps* = .05), whereas they did not differ from faster runners when they had to monitor for neutral PM cues.

**Fig 3:**
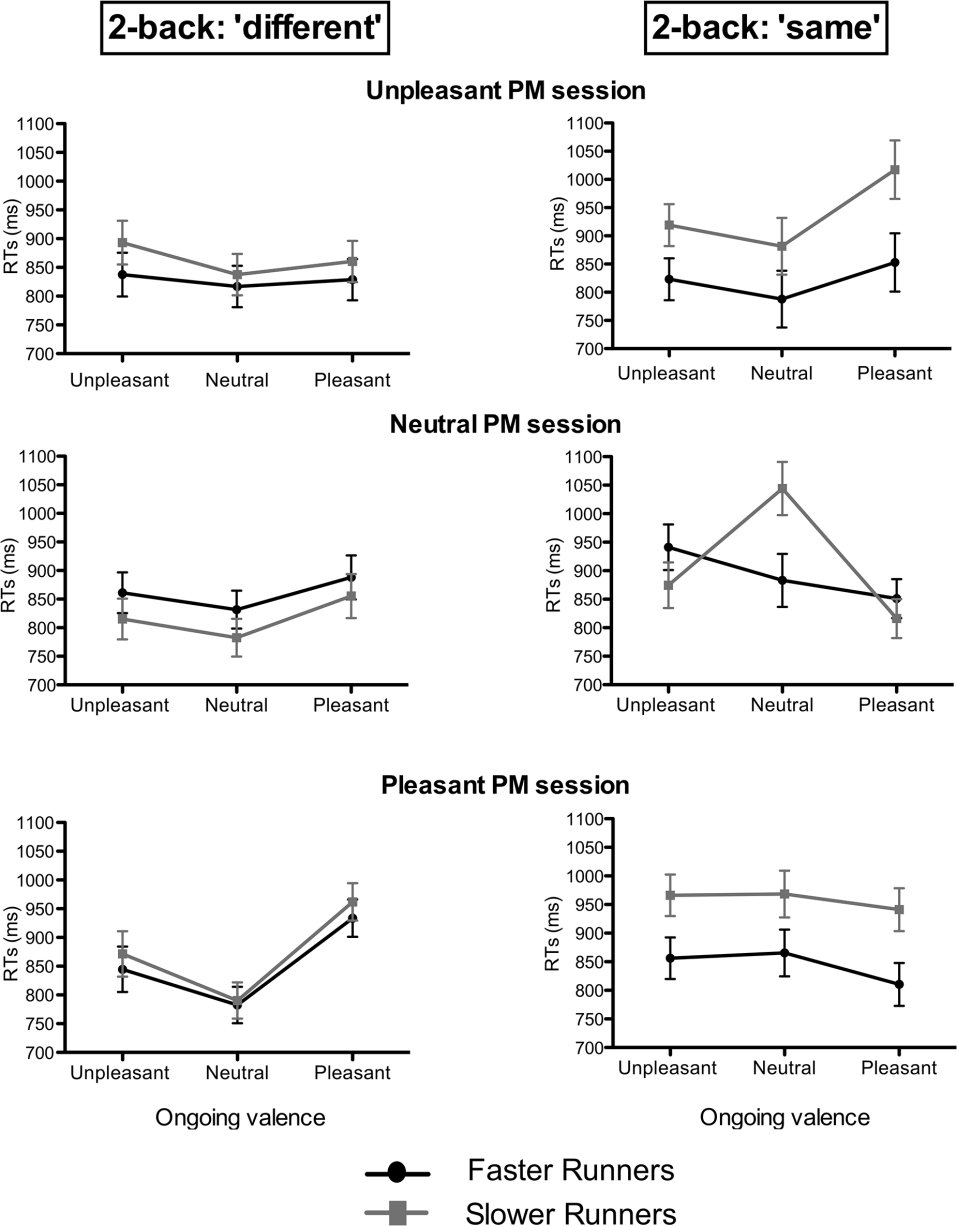
Mean reaction times (RTs) in the ongoing 2-back task, separately for each type of PM cue valence and ongoing stimulus valence. Runners group differences were observed in the ‘same’ trials, especially when participants had to monitor for unpleasant and pleasant PM cues and when the valence of the PM cue matched the valence of the ongoing stimuli.

The three-way and the four-way interactions were both significant. To better investigate the pattern of results in ongoing performance, the highest-level interaction [*F*(4,112) = 3.31, *p* < .01, *η*_*p*_ *²* = .10] was split in two ANOVAs, separately for the ‘same’ and the ‘different’ 2-back stimuli (Fig 3). Indeed, while there were not significant group differences in ‘different’ 2-back stimuli (all *ps* > .05), the effect of the Runners group was shown to be significant in the ANOVA performed on the ‘same’ stimuli (i.e., pictures that were also presented two stimuli before). More specifically, this analysis revealed a significant Runners group × PM cue valence × Ongoing stimulus valence interaction [*F*(4,112) = 4.23, *p* < .01, *η*_*p*_ *²* = .13]. As can be seen in Fig 3, if compared with the faster runners, the slower runners showed an increase in the RTs especially when the valence of the ongoing trials matched the valence of the PM cue to be monitored for in that session, thus revealing a higher SSIE. Indeed, the slower runners had slower RTs for pleasant ongoing pictures when monitoring for pleasant PM cues (p < .05), and for neutral ongoing pictures when monitoring for neutral PM cues (p < .01). The pattern of results in the unpleasant session was instead less clear, as slower runners showed increased RTs especially for pleasant ongoing stimuli (p < .01).

The analysis of the accuracy in the ongoing task did not reveal any significant effect (all *ps* > .05).

The analysis of the RTs in the PM task showed a significant interaction between Runners group and PM cue valence factors [*F*(2,56) = 3.37, *p* < .05, *η*_*p*_ *²* = .11]. Faster runners responded more quickly than slower runners to both pleasant PM cues (Faster runners: mean = 308 ms, SE = 32.97; Slower runners: *M* = 463 ms, *SE* = 49.49; *p* < .05) and unpleasant PM cues (Faster runners: *M* = 359 ms, *SE* = 39.25; Slower runners: mean = 496 ms, SE = 51.99; *p* < .05), whereas they did not differ between each other in responding to neutral PM cues (Faster runners: *M* = 414 ms, *SE* = 55.30; Slower runners: *M* = 422 ms, *SE* = 46.68; *p* > .05).

The analysis of the accuracy in the PM task did not show any significant effect (all *ps* > .05).

## Discussion

What are the factors that make an outstanding athlete? In the last decades it appeared clear that there is a combination of multiple factors, many of these are not strictly related to physical skills but concern other individual aspects, such as cognitive abilities. The present study corroborates this view, showing that some of the cognitive measures seem to be predictive of the quality of running performance.

More specifically, the findings indicate that, as compared with slower runners, faster runners had a better accuracy in nogo trials of the ICT, in which it was required to promptly inhibit a dominant, but inappropriate, response. Thus, our study showed enhanced motor inhibition in faster runners, suggesting that such cognitive function might be essential for successful running performance. Importantly, it extends the results of previous studies, which found comparable results on motor inhibition in soccer, baseball, tennis and volleyball players [15–17] so revealing that motor inhibition is crucial not only in team sports but also in endurance sports. As hypothesized by Verburgh et al. [31], motor inhibition might have a key role in some physical skills, as agility. Agility has been indeed defined as ‘‘a rapid whole-body movement with change of velocity or direction in response to a stimulus” [32]. We can speculate that the ability to re-direct a movement in response to a stimulus might be particularly important in a mountain ultra-marathon consisting in running and walking uphill and downhill in pebbly and stony terrains. Future studies might be useful to investigate whether motor inhibition has the same influence also on marathons that are performed on flat city roads. Conversely, no group difference was found for selective attention and working memory, which were evaluated in detect and go trials of the ICT. These abilities might be more required in other kinds of sports, as in soccer, baseball, or volley, which rely more upon strategic abilities as well as upon the execution of rapid actions towards stimuli.

Finally, the executive functions were evaluated in more depth by exploring the results on a particular kind of dual-task paradigm. In general, the performance to the 2-back working memory task did not vary according to the runners group. This would corroborate the findings obtained with the ICT in indicating that working memory has not a great importance on running. However, investigating the RTs in the 2-back trials depending on the valence of the PM cue to monitor for provided information about the interference derived from checking the presence of an emotional PM cue on ongoing task. To this regard, we observed that the interference on RTs due to the addition of the PM task was greater for the slower runners than the faster runners. More specifically, such greater interference shown by slower runners was observed in the RTs for the 2-back ‘same’ stimuli (i.e., stimuli that were same to those presented two trials before) and it was displayed in particular when individuals had to monitor for emotional PM cues. Notably, slower runners tended to have a greater Stimulus Specific Interference Effect (SSIE), which consisted of the increase in RTs when the valence of the PM cue matched the valence of the ongoing stimulus [25]. Therefore, a possible explanation is that slower runners were less able to suppress/inhibit the interfering representation of the PM cue, especially when such cue was emotional and when the task was more demanding (as in the ‘same’ trials). The increased SSIE for slower runners also supports this view, indicating that the group difference was observed mainly when the PM cue valence matched the ongoing stimulus valence, thus when the degree of interference between the internal representation of the PM cue and the external ongoing stimulus was higher given their similar valence. Following the Attention to Delayed Intention (AtoDI) model [26], our hypothesis is that faster runners tended to be more focused on external, ongoing stimuli, and were more effective at inhibiting the internal interfering PM cue representation. In this sense, outperforming runners seem to have a better inhibitory control not only over motor responses, but also over interfering distracting information. By contrast, slower runners tended to be more focused on the internal representation of the PM cue, which was less effectively inhibited. This finding is in agreement with the literature on the focus of attention, which highlighted that adopting an external focus of attention was associated with a better sporting performance and an increase in running economy [11–13; 20]. This was probably the experience that the writer Haruki Murakami meant to describe in the sentences that we reported at the beginning of the present manuscript. When he wrote “…*the kinds of thoughts and ideas that invade my emotions as I run remain subordinate to that void*” he might indeed refer to the tendency to focus on the ongoing activity as, in this case, on running, without being distracted by internal, momentaneously not relevant, thoughts.

An alternative hypothesis is that slower runners were more motivated to perform successfully the PM task, thus they were more engaged in monitoring for the presence of the PM cue amid ongoing stimuli. However, this would not explain why group difference was observed only in the 2-back ‘same’ stimuli and not in the 2-back ‘different’ stimuli. Furthermore, as compared with the faster runner, the slower runners took more time in executing the intention when the PM cue was emotional (i.e., pleasant and unpleasant). This seems to indicate that the emotional content of information had a greater impact on the motor responses in the slower runners, leading to the suggestion that the different way to process and react to emotional stimuli might contribute to account for differences in running performance [28–29;33].

A limitation of this research is that the race has been interrupted, thus one might wonder whether the rank at the intermediate gate would have been confirmed by the final rank. Basically, would this ranking have been somehow the same also at the end of the competition? Although we cannot answer this question, we sought to clarify this issue by analyzing the rank obtained by each runner in the last race that was characterized by a similar running distance, which was freely available on the web. Then, we compared the mean rank between faster runners and slower runners. We found that also for that race, the faster runners had a better running performance compared to the slower runners (Mean ranking score: Faster runners = 47.2 ± 40.5; Slower runners = 116.9 ± 87.5; *p* < .01). This suggests that the intermediate ranking of the Trans d’Havet race was likely to reflect the final ranking, so it was a good index to differentiate runners.

Finally, another question that can arise from this research concerns the role of cardiorespiratory fitness in modulating the cognitive performance of ultra-runners. It might be possible that faster runners had a better cognitive performance compared to slower runners since they were characterized by higher cardiorespiratory fitness. The relationship between cardiorespiratory fitness and cognitive efficiency has been indeed increasingly explored over the past decades, especially in relation to age-related cognitive differences [34–36]. Higher cardiorespiratory fitness was found to be associated with increases in white and grey matter volume in the prefrontal, parietal, anterior cingulate and temporal cortices and in the hippocampus, leading to improvements in multiple cognitive functions, such as attention, control and memory [34–38]. In the present experiment, we could not collect and assess physiological parameters, as maximal oxygen uptake (VO_2_max) and Running Economy (RE) because of logistic reasons (participants came from all over Italy and stayed at the experiment’s location only for the duration of the race). Nevertheless, we suppose that cardiorespiratory fitness played a minor role in accounting for the cognitive differences highlighted by the present research, for two main reasons. First, several studies showed that the VO_2_max is not a good performance predictor in homogeneous groups [39]—as our sample is—since it does not vary with great extent within such kind of groups. Second, we found that the cognitive differences between faster and slower runners involved selectively some functions (e.g., inhibition but not working memory and selective attention) rather than consisting in a global difference in cognitive functioning, which would be instead the expected result of variations in cardiorespiratory fitness. However, our hypotheses are still speculative, hence they need to be tested in future studies.

Summarizing, this is the first study to highlight that cognitive functioning seems to be predictive of the quality of running performance in ultra-trail. Indeed, as compared with slower runners, outperforming runners have a better inhibitory control, showing superior ability not only to inhibit motor responses but also to suppress processing of irrelevant distracting information. Their cognitive performance also seems to be less influenced by emotional stimuli. This research might open new directions toward understanding what cognitive and emotional factors characterize talented runners.
